# Research Progress on the Mechanism of the Acupuncture Regulating Neuro-Endocrine-Immune Network System

**DOI:** 10.3390/vetsci8080149

**Published:** 2021-07-30

**Authors:** Jingwen Cui, Wanrong Song, Yipeng Jin, Huihao Xu, Kai Fan, Degui Lin, Zhihui Hao, Jiahao Lin

**Affiliations:** 1 College of Veterinary Medicine, China Agricultural University, No. 2, Yuanmingyuan West Road, Haidian District, Beijing 100193, China; cuijingwen@cau.edu.cn (J.C.); 18604583203@163.com (W.S.); yipengjin@cau.edu.cn (Y.J.); xuhuihao2dai@163.com (H.X.); dirtyfan@163.com (K.F.); bs20193050484@cau.edu.cn (D.L.); 2 Center of Research and Innovation of Chinese Traditional Veterinary Medicine, Beijing 100193, China

**Keywords:** acupuncture, neuro-endocrine-immune network, mechanism, bidirectional regulation, microenvironment

## Abstract

As one of the conventional treatment methods, acupuncture is an indispensable component of Traditional Chinese Medicine. Currently, acupuncture has been partly accepted throughout the world, but the mechanism of acupuncture is still unclear. Since the theory of the neuro-endocrine-immune network was put forward, new insights have been brought into the understanding of the mechanism of acupuncture. Studies have proven that acupuncture is a mechanical stimulus that can activate local cell functions and neuroreceptors. It also regulates the release of related biomolecules (peptide hormones, lipid hormones, neuromodulators and neurotransmitters, and other small and large biomolecules) in the microenvironment, where they can affect each other and further activate the neuroendocrine-immune network to achieve holistic regulation. Recently, growing efforts have been made in the research on the mechanism of acupuncture. Some researchers have transitioned from studying the mechanism of acupuncture as a single linear pathway to using systems approaches, including metabolomics, genomics, proteomics and biological pathway analysis. This review summarizes the research progress on the neuro-endocrine-immune network related mechanism of acupuncture and discusses its current challenges and future directions.

## 1. Introduction

Acupuncture, originating in China, can be traced back to more than 2500 years. It is one of the most time-honored treatment methods and an indispensable component of Traditional Chinese Medicine. It is practiced by inserting and twisting acupuncture needles in multiple directions and speeds, aiming to cure diseases under the theory of traditional Chinese medicine. Currently, acupuncture has been accepted to a certain extent throughout the world. According to the traditional medicine strategy of the World Health Organization (WHO) (2014–2023) [1], 183 countries around the world have launched acupuncture projects. However, it is challenging to obtain a widespread understanding of its effect. The unclear mechanism of acupuncture impedes its extensive application. Compared with Western medicine, acupuncture is a physical stimulus that restores normal function by adjusting the internal environment and rebuilding physiological homeostasis, instead of directly acting on the pathogen. Besides, acupuncture is both holistic and bidirectional [2]. This also makes summarizing the mechanism of acupuncture based on a single factor or system impossible. The neuro-endocrine-immune network was not proposed until the 20th century [2]. Under the dominant control of the central nervous system, the body’s functions can be coordinated and regulated through the integration of the neuro-endocrine-immune networks. The body can then respond adaptively to the stimulation of the internal and external environment to maintain a steady state. Following this novel concept, the primary research into acupuncture has gone further. At present, several studies have reported that acupuncture can regulate the body’s function through adjusting the neuro-endocrine-immune network. Therefore, in this article, we review the relevant studies on acupuncture’s effects on the neuro-endocrine-immune network to find evidence that acupuncture regulates the neuro-endocrine-immune network and reveal the mechanism underlying acupuncture.

## 2. What Is the Neuro-Endocrine-Immune Network?

In 1977, Prof. Besedovsky [3] first proposed the theory of the “neuro-endocrine-immune network,” suggesting that there may be a common set of chemical information molecules and receptors among the neural, endocrine and immune systems. The intricate and complex interactions among these systems form a multi-dimensional network to maintain stability and improve body function. Subsequently, studies have shown that that the three systems work through feedback regulation.

The neuroendocrine system mainly affects the immune system by releasing neurotransmitters, neuropeptides and hormones. Corresponding receptors have been identified on immune cells, such as adrenergic receptors, cholinergic receptors, steroid receptors and growth hormone (GH) receptors [4,5]. Kawashima et al. demonstrated that both muscarinic and nicotinic acetylcholine receptors (mAChR and nAChR, respectively) expressed on lymphocytes can regulate the production of cytokines such as TNF-α, resulting in changes of antibody production [6]. In addition, the removal of the pituitary gland leads to the atrophy of lymphoid organs and the progressive destruction of systemic immune function. According to the effect of pituitary hormones on the immune system, they can be divided into the following two categories: the immune-enhancing hormones, including GH, prolactin (PRL), thyroid stimulating hormone (TSH), β-endorphin (β-EP), etc., and the immune-suppressive hormones, including β-EP, adrenocorticotropic hormone (ACTH), gonadotropin-releasing hormone (GnRH), somatostatin (SS), etc. [7,8]. The activated mononuclear phagocyte-hypothalamus-pituitary-adrenal (HPA) axis regulatory circuit is an example of endocrine immunoregulation. The corticotropin-releasing hormone (CRH) released by the hypothalamus promotes the synthesis of glucocorticoids through the HPA axis. Glucocorticoids (GC) and ACTH can inhibit the function of mononuclear phagocytes and reduce the release of IL-1, respectively. Increased production and release of IL-1 by activated mononuclear phagocytes will affect the hypothalamus and then stimulate the release of GC from the adrenal cortex. In turn, the release of IL-1 is inhibited. Melatonin (MLT) released by proopiomelanocortin (POMC), the precursor of ACTH, can counteract the IL-1 stimulation of hypothalamic secretion of CRH at the central level [9,10].

The immune system not only possesses receptors for neurotransmitters and endocrine hormones but also synthesizes and releases neurotransmitters and endocrine hormones, including acetylcholine, dopamine, TSH, GH, vasoactive intestinal peptide (VIP), PRL, etc. These functions play a role in local immune regulation and more complex neuro-endocrine-immune regulation circuits [11,12]. Moreover, Besedovsky [13] considers the immune system to be a sensory system, which receives information and converts it into appropriate signals and then sends signals to specific brain centers via humoral or neural pathways, producing a range of physiological responses sequentially. Cytokines are essential mediators in the humoral pathway. For example, IL-1 stimulates nerve cells to ingest glucose to support the metabolism and function of the central nervous system. Several cytokines are confirmed to affect the release of anterior pituitary hormones by acting on the hypothalamus and/or the pituitary gland. The major cytokines involved are IL-1, IL-2, IL-6, IL-18, TNF-α and interferon (IFN). The predominant effects of these cytokines are to stimulate the HPA axis and to suppress the hypothalamic-pituitary-thyroid (HPT) and gonadal axes and GH release [14,15].

## 3. Brain Regions Associated with the Neuro-Endocrine-Immune Network

Research indicates that some brain regions are associated with the neuro-endocrine-immune network.

The hypothalamus is known to be crucial for both cellular immunity and humoral immunity. The anterior hypothalamus (AH) is one of the vital regulatory regions. It has been reported that the electric stimulation of AH may up-regulate plasma cortisol and epinephrine levels reversibly and cause a decrease in the total lymphocyte counts [16]. In addition, studies have indicated that the electric lesion of AH induces a reduction in splenic natural killer (NK) cells’ activity, which is affected by pituitary factors [17]. The electric lesion of AH can lead to changes in the cell structure of immune organs and affect the production of antibodies, which subsequently affect cellular and humoral immunity.

The paraventricular nucleus of the hypothalamus (PVN) is associated with the regulation and release of various hormones, such as oxytocin, vasopressin, corticotropin-releasing hormone and thyrotropin-releasing hormone (TRH). Hefco et al. [18] showed that PVN lesion causes a decrease in the total leukocyte and lymphocyte counts and activity but increases cellular immunity mediated by T lymphocytes. Attributed to the thyrotropin-releasing hormone released by PVN, the lesion of the latter significantly reduces the level of T4 and T3 in plasma, which creates a stimulating effect on immune response in vivo. Based on the results, PVN influences the immune system via its neuroendocrine secretions and by activating the sympathetic nervous system.

Both the lateral hypothalamus (LH) and the anterior amygdala (AA) are part of the reward pathway, which is related to natural behaviors and emotions, and even drug addiction and withdrawal. Studies show that the electric stimulation of the reward pathway may lead to similar effects with natural stimulation, such as eating, drinking and feeding behavior. In addition, researchers have found that the electrical stimulation of the LH brain region plays an immunomodulatory role. Tsuboi et al. [19] showed that LH lesion resulted in a decrease in splenic cells and an increase in the apoptosis of splenic lymphocytes. It indicated that LH could affect cellular immunity by apoptosis in the spleen. Other studies have revealed that the expression of TGF-β, Caspase3, and some other cytokines are associated with immune function regulated by LH and AA [17].

The limbic system, involved in learning, memory, emotion, and regulating visceral activity, is composed of the limbic lobe and subcortical structures closely related to it, such as the amygdala and hippocampus. The hippocampus is a falciform arch located at the inferior angle of the lateral ventricle, which plays an essential role in learning and memory. The amygdala is located in the dorsal medial part of the anterior temporal lobe, the slightly anterior position of the hippocampus, and the apex of the inferior horn of the lateral ventricle. It has been reported that the hippocampus and amygdala could regulate the hypothalamic-pituitary-adrenal axis through both neural and immune responses. Both the hippocampus and amygdala have nerve fibers projected into the hypothalamus and into each other. The effects of IL-2, IL-16 and TNF on the hippocampus and amygdala suggest that they are both affected by the immune system. In contrast, the hippocampus and amygdala are involved in regulating immune function [20]. Damage to the hippocampus can affect humoral immunity, while damage to the hippocampus or amygdala can control the number of cells in the immune organs [21]. Thus, some structures of the limbic system are intimately associated with neural and immune regulation.

## 4. Physiological Significance of Acupoints

It is well known that acupoints are the way that acupuncture acts on the body. Acupoints are considered to be specific points that reflect the status and regulate the function of the *Zang-Fu* and are also an important link for the relationship between meridians and *Zang-Fu*. Clinical observations and primary research have indicated that the adjustment of *Zang-Fu* by acupuncture depends on the neuro-endocrine-immune network to a large extent [22]. Many animal and human studies have shown that the needling of acupoints can cause a variety of biological reactions, which can occur locally or at a distance. This distant effect is thought to be mediated primarily by sensory neurons, which project through afferent nerves onto a series of structures of the central nervous system and affect various physiological systems [22] (Table 1 for specific examples). Electrophysiological experiments have demonstrated that acupuncture can activate baroreceptors, stretch receptors and free nerve endings at acupoints [23]. Peripheral sensory fibers are divided into four categories according to their diameter, all of which can be excited by hand needles, and their main functions are summarized in Table 2. The observation of direct wound stimulation in trauma patients showed that the spurs on the nerve trunk were mostly numb, while those on the blood vessels were mostly aching, those on the tendon periosteum were most sore, and those on the muscles were mostly swollen [24]. Different sensations activate different nerve fibers. The numbness mostly excites the type II fibers, the swelling pain mostly excites the type III fibers, and the soreness mostly excites the type IV fibers. Moreover, different sensations induce different responses in the brain’s regions, which secrete various chemical mediators to regulate endocrine and immune functions [25].

Additionally, some recent studies have indicated that acupoints are distributed in areas of neuroimmune modulation. Histologically, acupoints are also found in high-density regions of mast cells, lymph-vessels and arteriovenous plexuses in addition to areas with concentrated innervation [37]. Immunohistochemistry and fluorescence microscopy has proven synaptic connections between mast cells and nerve endings [38]. Furthermore, the western blot test confirmed that transient receptor potential vanilloid subtype 2 (TRPV2) channel protein expression on the mast cell membrane. This channel could be activated by mechanical force and high temperature [39]. Thus, acupuncture or moxibustion can stimulate mast cells’ degranulation and release of histamine, substances P and 5-HT, etc. In addition to increasing vascular permeability, these biological media amplify the effect through axonal reflexes and neural interactions.

## 5. Characteristics of Acupuncture

Acupuncture is a kind of physical stimulation that can stimulate or induce the function of the internal regulation system and restore the physiological and biochemical processes to normal. Acupuncture fully exploits its stimulation effect on the physiological and pathological processes of the body and the reaction of these effects in vivo to regulate the organism. The characteristics of acupuncture include bidirectional and benign regulation, holistic and comprehensive regulation, self-limiting regulation and quality regulation [40,41].

Bidirectional regulation refers to the fact that acupuncture points can produce bidirectional effects, such as excitation or inhibition. The direction of action depends on the functional state of the body. Acupuncture at the Tianshu (ST25) acupoint can both relieve constipation and treat diarrhea [42]. In addition, many other bidirectional regulation features of acupuncture embody similar therapeutic effects of promoting sweating and inhibiting sweating, raising and lowering blood pressure and treating urinary retention and incontinence.

Benign regulation means that after acupoint stimulation, the body’s pathological state generally changes to its normal physiological state. The bidirectional and benign regulation characteristics of acupuncture are fundamentally different from the effect of drugs. The application of acupuncture can produce curative effects with barely any adverse reactions. With the exception of injury attributed to improper technique, acupuncture generally will not cause damage to the body [43].

Holistic and comprehensive regulation means that acupuncture can regulate the body at multiple levels and targets, including the multi-target regulation of the same organ and multi-organ or multi-system regulation of the body’s overall state, which is the fundamental reason for the extensive indications of acupuncture. For instance, the treatment of irritable bowel syndrome by acupuncture is the multi-target regulation of the same organ, as mentioned above. Its treatment involves a variety of active substances such as cholecystokinin and vasoactive intestinal peptide, which are involved in the regulation of gastrointestinal function at different levels [44].

The self-limiting regulation of acupuncture can be illuminated from two aspects. Firstly, the regulating ability of acupuncture is limited and can play a role only within the normal range of physiological regulation. Secondly, the regulating capacity of acupuncture depends on the integrity of the relevant tissue structure. Under normal circumstances, the intensity of a drug’s therapeutic effect increases with an increase of drug dose, which will eventually result in off-target effects. However, the regulating effect of acupuncture presents a saturation characteristic, that is, the self-limited regulation of acupuncture [45].

Quality regulation refers to the function of acupuncture to improve the quality of the internal regulation system and enhance the ability of the organism’s self-regulation to maintain the stability of the whole internal environment. This aligns with “neuro-endocrine-immune network” theory. For example, the Zusanli (ST36) acupoint can invigorate the spleen/stomach and replenish Qi through the Stomach Meridian of Foot-Yangming. Acupuncture at this acupoint can relieve pain, regulate lipid metabolism and enhance the body’s immunity, which plays a vital role in healthcare so as to reflect the quality regulation of acupuncture [46].

Broad applications with a low risk of adverse reactions are the advantages of acupuncture, which are closely related to its characteristics. To develop research ideas and to improve its curative effect, it is important to understand the characteristics of acupuncture.

## 6. Mechanisms of Acupuncture on the Neuro-Endocrine-Immune Network

### 6.1. Effects of Acupuncture on the Nervous System

Acupuncture can regulate the release of neurotransmitters, neuropeptides and hormones by stimulating the neuroendocrine system. In addition, acupuncture can also indirectly affect the immune system by regulating the neuroendocrine system, specifically embodying the substances released by the neuroendocrine system acting on the corresponding receptors of immune organs and immune cells.

#### 6.1.1. The Effects of Acupuncture on Neurotransmitters

As benign stress, acupuncture can change the concentration of certain neurotransmitters, including monoamines and acetylcholine.

As the main neurotransmitters of sympathetic nerves, the concentration of monoamine neurotransmitters in the blood can be increased significantly by sympathetic nerve excitation, thereby affecting the immune system. Catecholamine (CA) has been shown to inhibit the synthesis of pro-inflammatory factors such as IL-12, IFN-γ and TNF-α and to up-regulate the expression of anti-inflammatory factors such as IL-10 and TGF-β [47]. As an indoleamine, 5-hydroxytryptamine (5-HT) is not only an important neurotransmitter but also a vital immunomodulatory factor. It can act directly on immune cells and regulate a variety of immune functions through receptors or other channels.

Studies have shown that acupuncture has a regulating effect on monoamine neurotransmitters. Acupuncture at DU-16 can improve the memory impairment of dementia mice by significantly increasing the contents of 5-HT, norepinephrine (NA) and dopamine (DA) in the brain tissue [48]. For intracerebral hemorrhagic rats with increased release of CA, acupuncture at PC-6, GV-26 and ST-9 can inhibit the release of CA [49]. Studies have proven that electroacupuncture can reduce the levels of the plasma monoamine neurotransmitters 5-HT, DA and NE to relieve anorexia in rats [50]. As narrated above, the effects of acupuncture on various diseases are related to the regulation of monoamine neurotransmitters.

Acetylcholine is the main neurotransmitter of the vagus nerve, which regulates the production of cytokines on a variety of immune cells by activating α7 nicotinic acetylcholine receptors (nAChRs) and promoting T cell development and/or differentiation [51]. Both nAChRs and muscarinic acetylcholine receptors (mAChRs) can regulate the synthesis and release of cytokines such as TNF-α, IFN-β and IL-6, thereby regulating immunity [6].

Studies have shown that acupuncture can increase the low level of acetylcholine in patients with Parkinson’s disease and dementia as well as promote the production of acetylcholine in intracerebral hemorrhage rats with reduced acetylcholine release [52]. According to the research, acupuncture at GV-20 and K-11 in the treatment of Alzheimer’s disease resulted in increased levels of plasma acetylcholine, which is one of the biological mechanisms [53]. In addition, the mechanism of acupuncture at BL-13 in the treatment of allergic asthma in rats is also related to the inhibition of the synthesis and release of acetylcholine [54]. Therefore, whether acupuncture can affect the immune system by regulating acetylcholine is worth exploring further.

#### 6.1.2. The Effects of Acupuncture on Neuropeptides

Neuropeptides, the regulatory substances of the nervous system, are one of the important mediators in the regulation of the neuro-endocrine-immune network system. It mainly includes opioid peptides and brain-gut peptides.

Opioid peptides comprise endorphins, dynorphins and enkephalins. They are opioid-active peptides in the brain that function through opioid receptors. The effect of opioid peptides on immune function by binding to atypical opioid receptors on the surface of lymphocytes has been demonstrated. It can regulate lymphocyte transformation, the E-rosette test for T lymphocytes, NK cell activity, macrophage function, interferon production, etc. [55,56]. Endogenous opioid peptides were first found to have a similar effect to morphine, which alleviate pain and reduce sympathetic excitation [57]. Among the opioid peptides, endorphins are mainly found in the brain and pituitary. Representatively, β-endorphins (β-EP) can regulate the secretion of the adrenocorticotrophic hormone (ACTH) and ease pain [57]. In vitro experiments by Prezew et al. [58] revealed that β-EP could enhance the function of a variety of immune cells. β-EP and dynorphins can enhance the release of oxygen free radicals by macrophages and polymorphonuclear cells, thus enhancing the killing effect on tumor cells [59]. Moreover, enkephalins include methionine enkephalins (MENK) and leucine enkephalins (LENK). The regulatory effect of MENK on the immune system is reflected in many aspects, such as regulating macrophage phagocytosis and phenotypic polarization [60], activating NK cells and enhancing their activity [61,62,63,64], promoting the maturation of dendritic cells within a certain concentration range [65], regulating the growth of B lymphocytes bi-directionally [66] and affecting the release of cytokines [67]. Furthermore, studies on the mechanism of acupuncture’s effect on the immune activity of NK cells have found that LENK can increase the lethality of NK cells [59].

Studies have shown that acupuncture can affect the synthesis, release and degradation of opioid peptides. Dong et al. [68] demonstrated that acupuncture could promote the central secretion of opioid peptides in patients with headaches in the recovery phase of ischemic stroke to reduce the pain stress response and the secretion of pain-related factors, thus playing a therapeutic role. Moreover, acupuncture can promote the repair of gastric mucosal injury and improve gastrointestinal function, which is related to the decrease of β-EP in plasma and the increase of β-EP in the hypothalamus [69]. In addition, research has shown that after acupuncture at PC-6, the content of LENK increased in the cauda nucleus, the hippocampus and thalamus, the pituitary and plasma. This suggested that the synthesis and release of opioid peptides were increased in different brain regions, the pituitary, and other tissues during acupuncture [59]. Furthermore, a study on dynorphin-A (DYN-A) participating in electroacupuncture in the treatment of exercise-induced central fatigue has indicated that electroacupuncture can regulate the level of DYN-A in the nuclei nervorum cerebralium during exercise fatigue and maintain the dynamic stability of DYN-A so as to make the body better adapt to the exercise intensity [70]. Therefore, despite the lack of relevant research, we reasonably suspect that acupuncture can mediate immune regulation by regulating opioid peptides.

Brain-gut peptides consist of substance P (SP), vasoactive intestinal peptide (VIP), cholecystokinin (CCK), etc. SP is an immunomodulatory peptide that can promote the differentiation and proliferation of B lymphocytes, increase the synthesis and secretion of antibodies, and obviously promote the phagocytosis of mononuclear macrophages [59]. Moreover, it can enhance the activity of NK cells and promote their secretion through the NK1R receptor, which is related to the MAPK signaling pathway [71]. VIP is a widely distributed neuropeptide. It exerts an immunomodulation effect by interacting with specific receptors mainly in the following ways: on one hand, VIP inhibits the expression of inflammatory cytokines (such as TNF-α, IL-6, and IL-12), increases the expression of anti-inflammatory cytokines (such as IL-4 and IL-10) [72] and inhibits the expression of inflammatory chemokines and adhesion molecules [73]. On the other hand, it regulates the Th2/Th1 balance, promotes the production of regulatory T cells (Tregs) and inhibits the effect of T helper cell 17 (Th17), thereby exerting immunosuppressive and anti-inflammatory effects [74,75,76,77]. This anti-inflammatory mechanism has been demonstrated in experimental autoimmune encephalomyelitis (EAE) rats [78]. As for CCK, it can regulate immune function by influencing lymphocyte DNA synthesis, the adhesion and chemotaxis of immune cells, the phagocytic function of macrophages and the secretion function of immune cells [79,80].

Several studies have demonstrated that the therapeutic effects of acupuncture are related to the regulation of brain-gut peptides. Initially, SP has different physiological effects on the body. β-EP acts as one of the markers of changes in the body. It often predicted reduction in headache frequency when β-EP level above 4 ng/mL in cerebrospinal fluid [81]. In contrast, SP is a pain-related excitatory transmitter, which will further aggravate the pain if its concentration increased. As previously mentioned in the treatment of patients with headache in the recovery phase of ischemic stroke, acupuncture, in addition to promoting the secretion of opioid peptides, can also reduce the contents of pain-related factors SP, DA and 5-HT in plasma, thus achieving an analgesic effect [68]. At present, the analgesic effect of acupuncture by inhibiting the release of SP has been confirmed by several studies, such as in the treatment of ischioneuralgia, knee osteoarthritis, lumbar disc herniation and other diseases [82,83,84]. The effect of electroacupuncture therapy in rheumatoid arthritis can be achieved by up-regulating the local VIP expression level. Its mechanism has already been described [85]. Additionally, VIP plays a certain regulatory role in the proliferation, differentiation and maturation of thymocytes. In the acupuncture therapy of sepsis rats, it has been found that acupuncture at ST-36 and CV-4 can improve the level of VIP in the pituitary and peripheral blood to reduce thymocyte apoptosis [86]. Ultimately, studies on acupuncture’s regulation of CCK level are mostly concerned with its physiological effects, that is, promoting gastrointestinal secretion and facilitating the contraction of the gallbladder. It is speculated that acupuncture at ST-36 regulates the expressions of Ghrelin, VIP, and CCK and its receptors in rats, thus regulating the syndrome of Spleen Qi deficiency [87]. In conclusion, the therapeutic effect of acupuncture is possibly related to the regulation of brain-gut peptides.

### 6.2. Effects of Acupuncture on the Endocrine System

In this section, the hypothalamic-pituitary-thyroid axis (HPT axis) has been selected as an example, introducing the regulating effect of acupuncture on the endocrine system.

The HPT axis is the set point for the synthesis and release of the thyroid hormone (TH). The hypothalamic thyrotropin-releasing hormone (TRH) enhances the synthesis and secretion of the pituitary thyroid-stimulating hormone (TSH). It acts on the thyroid and stimulates all steps of TH synthesis and secretion, which is the result of the positive input of hypothalamic TRH and the negative input of circulatory TH. TH affects normal growth, metabolism, nutrient absorption and temperature regulation [88]. Additionally, TH is the fundamental regulator of the thyroid’s growth and function. At present, there has been a mass of reports on the acupuncture treatment of thyroid-related diseases. Acupuncture has a two-way regulating effect on HPT axis-related hormones. Therefore, acupuncture is effective for the treatment of hyperthyroidism and hypothyroidism [89].

There is also a multitude of evidence that TH is essential for the development of the nervous system. It can upgrade the formation of myelin sheaths, axon branching and synapse and cell differentiation and migration. It also counteracts the apoptosis of granulosa cells and regulates the development of Purkinje cells to form and maintains the integrity of the central nervous system [90]. Wei et al. [91] found that providing exogenous thyroxine after traumatic brain injury can promote the regeneration of the central nervous system significantly, which may be associated with the up-regulation of the expression of mRNA and increase the secretion of brain-derived neurotrophic factor (BDNF) and nerve growth factor (NGF).

In addition, the HPT axis may be related to the immune system through cytokines. Recent research has shown that TH could induce Th1/ Th2 cell imbalance through influencing cytokines, thus affecting the immune system. Hwang et al. [92] observed cytokine changes in Sprague-Dawley (SD) male rats after the intraperitoneal injection of L-thyroxine (LT4, 0.5 mg/kg) once a day for four weeks to simulate hyperthyroidism. Later, the effect of acupuncture on the process was observed after acupuncture treatment. The results showed that IFN-γ secretion increased significantly, while IL-4 secretion decreased considerably. However, after the acupuncture treatment, the secretion of IFN-γ decreased, and the secretion of IL-4 was increased, thus showing the effect of controlling Th1/Th2 immune imbalance. Finally, it was confirmed that this effect was related to the decrease of Foxp3 gene expression.

### 6.3. Effects of Acupuncture on the Immune System

As mentioned above, acupuncture can affect the immune system by regulating the neuroendocrine system. Meanwhile, acupuncture is also able to influence the neuroendocrine system by modulating the immune system. A large number of studies have shown that acupuncture has a benign regulatory effect on the immune system, involving innate immunity and acquired immunity.

#### 6.3.1. The Effects of Acupuncture on Innate Immunity

##### Immune Cells

Innate immunity consists of tissue barriers, immune cells and immune molecules. Acupuncture performs a two-way regulatory effect on most of them. The regulatory effects of acupuncture on mast cells have been discussed above and will not be repeated here.

Regarding macrophages, the effect of acupuncture on them is not obvious under normal physiological conditions. However, acupuncture can significantly enhance phagocytosis in pathological conditions or hypoimmunity. When the phagocytosis is hyperactive, acupuncture will down-regulate it to reduce the phagocytosis index. Current studies suggested that the regulatory effect may be associated with the activation of the cholinergic anti-inflammatory pathway (CAP) as well as the regulation of scavenger receptors’ (SRs) expression and M1/M2 macrophage polarization [93,94].

Acupuncture also has a benign regulatory effect on NK cells. Its mechanism is to regulate the expression of NK cell receptor CD94, protein tyrosine kinase (PTK), adhesion molecule VCAM-1, protein tyrosine phosphatase (PTP) and SH2-containing tyrosine phosphatase 1 (SHP-1) genes [95]. Moreover, acupuncture can stimulate the HPA axis to release endorphins that bind to the opioid receptors on the surface of NK cells and stimulate NK cells to increase the expression of cell adhesion molecules, granzyme B and perforin. Meanwhile, acupuncture intervenes in the regulation procedure of immunosuppression through NK cells. Studies have confirmed that the immune regulatory network, which is mainly composed of IL-2, IFN and NK cells, plays a crucial role in enhancing and regulating the body’s immune function, which is inseparably intertwined with some pathological conditions such as tumorigenesis [96].

Moreover, acupuncture can exert brain-protective effects by regulating microglia. Microglia are glial cells, which are another kind of immune cell mainly located in the central nervous system. They are the first and most vital defense in the central nervous system. In the healthy brain, microglia work hard as “logistical cells” to clear dead cells and generate neurotrophic factors that immerse neurons in protective factors. A large number of clinical studies have proven that activated microglia play a dominant role in the pathogenesis of neurodegenerative diseases, such as Parkinson’s disease, Alzheimer’s disease and multiple sclerosis [97]. However, under pathological conditions (such as cerebral ischemia), activated microglia will directly contact nerve cells and perform phagocytosis, resulting in brain damage. In addition, microglia cells will initiate the translocation of intracellular transcription factors into the nucleus to regulate downstream gene expression. This leads to the release of proteolytic enzymes, such as MMP-9 and inflammatory cytokines, such as IL-1β, IL-6 and TNF-α, eventually aggravating neuronal damage. Previous studies have shown that acupuncture can intervene in this mechanism [98,99]. Electroacupuncture at the GV-20 acupoint can lower the expression of TNF-α, IL-1β and IL-6 in central immune cells while also enhancing the expression of IL-10 through stimulating the sympathetic nerve and HPA axis, promoting microglia polarized toward M2 type and resulting in the increased secretion of neurotrophic factors [100]. Moreover, the activation of microglia will be restrained after electroacupuncture at the GV-20 and GV-16 acupoints, inhibiting the TLR 4/NF-κ B signaling pathway. The secretion of inflammatory mediators such as MMP-9, TNF-α, IL-1β and IL-6 will decrease afterward [99].

##### Immune Factors

Immune factors include lysozyme, defensins, complement, cytokines, etc., most of which can be bi-directionally regulated by acupuncture. Currently, it is known that immune factors are involved in the regulation process of acupuncture in most diseases. As for immune factors, numerous studies have confirmed that cytokines are key mediators in the neuro-endocrine-immune network [101]. Furthermore, cytokines’ diverse function is embodied in the mediation of multiple functions in non-immune tissues (such as the central nervous system, endocrine system, etc.) [102]. For example, Del Rey et al. [103] found that the IL-1 produced by astrocytes or neurons stimulates the transport of glucose from one cell type to another, suggesting that this cytokine can mediate the mutual energy supply between nerve cells. Meanwhile, cytokines such as IL-1 and IL-6 produced by the hypothalamus are involved in learning and memory consolidation, and the mechanism may be associated with synaptic plasticity [104]. Cytokines can also act directly on endocrine organs. Previous studies have demonstrated that topical IL-1 can induce GC secretion in the adrenal glands of anesthetized mice with hypothalamus excision [105]. The regulation mechanism of acupuncture on immune factors is mainly enacted through affecting the inflammatory response signaling pathway (such as nuclear factor-kappa B, p38 mitogen-activated protein kinase, extracellular signal-regulated protein kinases, etc.) and the surface receptors of immune cells or non-immune cells (such as toll-like receptor 4, complement receptor 1, cannabinoid receptor 2, etc.) [106,107,108]. According to different diseases, acupuncture bidirectionally regulates the generation of immune factors, forming a complex network of mutual influences to regulate physiological functions.

#### 6.3.2. The Effects of Acupuncture on Acquired Immunity

Previous studies of acupuncture on cellular immunity regulation established that it can regulate the number of T lymphocytes and their subsets, adjust the conversion rate of T lymphocytes, and also modulate the ratio of CD4/CD8 [109]. One of the primary mechanisms is the balanced tuning of Th1/Th2. In allergic rhinitis, bronchial asthma and chronic fatigue syndrome, acupuncture can reverse the Th1/Th2 balance in the direction of Th1 [110,111,112]. Conversely, while in depression and embryo implantation disorders, acupuncture can move the Th1/Th2 balance toward Th2 [113,114]. With the modulation of Th1/Th2, the cytokines secreted affect the Treg cells that play an indispensable role in maintaining the homeostasis of the immune environment [115]. However, under several pathological conditions, the balance will be jeopardized. Endogenous TGF-β and inflammatory mediators IL-6 or IL-21 induce the production of the retinoid-related orphan receptor γt (RORγt), which triggers Th0 cells to differentiate toward Th17 cells and generates an immune response. Then the impact of RORγt is inhibited with declining inflammatory mediators such as IL-6. Th0 cells are transformed into Treg cells simultaneously to stabilize the function of Treg [116,117,118]. The mechanism, together with previous clinical observations, led us to recognize the effect of acupuncture on the treatment of chronic obstructive pulmonary disease, depressive disorder, rheumatoid arthritis, etc. [119,120,121]. Moreover, acupuncture is probably conducive to amplifying the apoptosis rate of T cells in peripheral blood and impacting T cell tolerance by regulating the expression of FAS/FAS-L. The interaction of the transmembrane protein FAS and its ligand can initiate apoptosis in the thymus to alleviate rheumatoid arthritis [122].

Humoral immunity is an immune mechanism based on the production of antibodies by plasma cells to safeguard the body. Acupuncture also performs two-way modulation on B cells, which are responsible for humoral immunity. Wang et al. [123] indicated that acupuncture at CV-8 and ST-36 could increase the levels of IgG, IgM and IgA significantly in the serum of the elderly. Apart from this, acupuncture can decrease the concentration of antibodies in the serum if certain antibodies are overexpressed. As an example, Zhao et al. [124] found that the combination of acupuncture and immunosuppressive agents for the treatment of paraneoplastic syndrome can synergistically decrease the level of autoimmune antibodies, correct immune disorders, and improve electromyogram and nerve damage in patients. However, the author has found that there are inadequate studies on how acupuncture affects B lymphocytes directly to impact immunoglobulins, and relevant mechanisms need to be explored. Nonetheless, acupuncture can affect humoral immunity indirectly by influencing the release of antigen-presenting cells, cytokines, complements, etc.

At the end of the last century, the novel concept of “acupuncture serum” was advocated, which refers to the serum collected from the human or animal body after acupuncture treatment. The serum is appended to another system as an effective substance and then observed for its effect after contact with the organ, cell, or molecule in vivo or in vitro [125]. There is an assortment of bioactive substances contained in acupuncture serum, such as antibodies and immune factors, which all have an important influence on the nervous system, immune system, cardio-cerebral vascular system, etc. Acupuncture results in the release of cytokines, hormones and neuromodulators and neurotransmitters in the blood and these can have physiological effects on cells and tissues [126,127].

#### 6.3.3. The Effects of Acupuncture on the Skin Barrier

Recent studies have shown that skin is the largest immune organ of the body by not only having non-specific immune defense functions but also participating in the entire process of specific immune and skin immune responses. The skin immune barrier consists of cellular components such as keratinocytes, T/B lymphocytes, langerhans cells, endothelial cells, mast cells, macrophages, etc. and molecular components such as cytokines, immunoglobulins with specific defense effects, complements with non-specific immune functions, etc., as well as neuropeptides such as substance P (SP), calcitonin gene-related peptide (CGRP), etc. [128]. These components located in the skin barrier cooperate with the innate immune system inside the organism together to maintain the stability of the skin microenvironment and internal environment. Additionally, the skin is considered to be a neuroendocrine organ. Abundant studies have demonstrated that hair follicles and sebaceous glands have well-established HPA axial functions in response to mental stress and environmental stimuli [129,130].

The current research about acupuncture’s effects on local skin immunity mainly focuses on the interaction between immune cells, immune molecules, free nerve endings, the activation of the nervous system and local HPA axis, etc. In terms of the research on the effects of acupuncture on psoriasis, Wang et al. [131] demonstrated that electroacupuncture improved skin lesions, decreased epidermal thickness, inhibited the proliferation of keratinocytes, and reduced CD3 T cell infiltration significantly. In addition, electroacupuncture decreased the secretion of inflammatory cytokines, including IL-1β, IL-17A and IL-23p40, and down-regulated the expression level of neurokinin A (NKA), which is correlated positively to the extent of decreased inflammatory cytokines in local lesions [131]. According to the pertinent literature and ‘physiological significance of the acupoints’ mentioned above, we hypothesize certain routes after acupuncture into the skin: (1) Acupuncture will stimulate peripheral nerve endings and some receptors on the skin, and then the generated electrical signals are transmitted to the corresponding brain regions through the neural pathway, thereby regulating physiological functions. (2) Acupuncture creates a micro-traumatic environment in the pinhole, recruiting mast cells, macrophages, and other local cells to release certain biomolecules, such as histamine, 5-HT, SP, β-EP, etc. On the one hand, humoral immunity triggers regulation and induces subsequent acupuncture effects; on the other hand, this effect is amplified by axon reflexes and neural interactions. (3) Under the local stress stimulation of acupuncture, the skin HPA axis is activated, and then CRH, ACTH and cortisol are secreted sequentially. Since CRH performs dual-directional effects on anti-inflammatory and pro-inflammatory factors, the axis can regulate different physiological effects on different diseases through feedback mechanisms.

## 7. Deficiencies and Prospects of Acupuncture

Recent research has illuminated that the human body is a complex system, which is not a superposition of simple systems but rather formed by integrating different networks [132]. Acupuncture reveals the occurrence and development of diseases from a macro, holistic, and systematic perspective. It has been discovered that acupuncture is an effective physical stimulus that can act as a “promoter” to activate local cell functions and neuroreceptors, regulate the release of related chemicals in the microenvironment and how they affect each other, and further activate the neuro-endocrine-immune system. Therefore, it is challenging to summarize the mechanisms of acupuncture in previous studies using a single system. Increasing numbers of researchers are exploring the network and the complexity of the crosstalk between different systems. At present, the effect of acupuncture on various diseases has been confirmed by numerous studies, but most of them are statistically comparative studies. The depth and breadth of the research on its mechanism are still insufficient. The research level mostly stays on the therapeutic effects and cell level, while the research on the molecular mechanism has not yet been thoroughly explored. Meanwhile, the effects of acupuncture on the interaction between various cells and cytokines under the neuro-endocrine-immune system are also unclear. Recently, researchers have realized the close links between different systems and different organs, and acupuncture has performed a regulating effect on some key mediators of these systems. However, the specific mechanism of acupuncture on the interaction between these different systems has not been studied clearly. In addition, research on the transition from basic research on the functional mechanism of acupuncture to clinical practice is still scarce.

The development of systems biology and omics techniques has become a new trend in the network regulation and acupuncture therapy research. Several years of research have shown that acupuncture has immediate and delayed therapeutic effects. The immediate effect is mostly related to the signal transmission of the nervous system, while the subsequent effect is associated with the transmission of hormones, cytokines and other signals in body fluids. A variety of functional proteins can be produced through the body fluid pathways, which may lead to changes in proteomics. At the level of gene expression regulation, the transcriptomics technology can analyze the specific expression factors and biological mechanisms of acupuncture treatment against diseases. This will help to clarify the specific pathological and physiological regulation characteristics of acupuncture integrally. Metabolomics, starting from the final metabolites, traces the relationship between the metabolic changes of sugars, proteins and lipids in organisms and the occurrence and development of diseases. It can also track the time-effect relationship between the disease and acupuncture treatment as well as reflect the overall changes of the organism. Currently, with the advancement of metabolomics research, the metabolic targets of common clinical diseases treated by acupuncture are becoming clearer. The continuous improvements of the emerging analysis approaches of biological information, such as tracer technology, two-photon technology and cryo-electron microscopy technology, have also provided great convenience to acupuncture research. Therefore, the combination of the evolving omics technology and the emerging analysis methods of biological information may facilitate the study of acupuncture and enable the mechanism of acupuncture on the neuro-endocrine-immune network to be revealed intensively.

## Figures and Tables

**Table 1:** Examples of acupoints and its effects of regulating neuro-endocrine-immune network.

Acupoints	Pathway/Mediators Can Be Regulated	Regulating Effect	Reference
LI4	HA, 5-HT	Increasing mast cell degranulation rate, Increasing the concentration of HA and 5-HT, Demonstrating the connection between mast cells and nerve fibers.	[26]
GB20	BDNF, GFAP, NSE, IL-6, plasma ET	Reducing astrocyte and neuron damage, Lower blood pressure, Regulating cellular immunity and secretion functions of the vascular endothelial cells in hypertension patients.	[27,28]
GB20, GB34	CGRP, COX-2, BDNF, IL-1β, IL-6, TNF-α	Inhibition of dural mast cells, macrophages, and serum inflammatory factors, Alleviating hyperalgesia.	[29]
ST36, SP6	CRF, y-GABA, GAD	Accelerating the synthesis of GABA, Inhibiting CRF neurons, Inhibiting the overactivity of the HPA axis.	[30]
PC6, BL15	NE, DA	Decreasing the nerve electrical activity in spinal dorsal roots, Increasing the NE and DA concentrations in the paraventricular nucleus of the hypothalamus.	[31]
GV20, ST36	TNF-α, IL-1β, IL-6, 5-HT, CRH	Up-regulation of 5-HT1 receptor, Down-regulation of 5-HT2 receptor, Reducing the concentration of serum IL-1β and IL-6, the hypothalamic index and CRH mRNA decreased significantly, Relieving depression and chronic fatigue.	[32,33,34]
CV4, SP6	E2, FSH, LH, β-EP	Increasing E2 content, Reducing the contents of FSH and LH, Increasing the content of β-EP in hypothalamus, Regulating reproductive endocrine and autonomic nervous dysfunction	[35]
EX-B2	NF-κB pathway	Inhibiting NF-κB expression and activation, Inhibiting NF-κB signal transduction pathway through neuro-endocrine-immune network.	[36]

**Table 2:** Classification of peripheral sensory fibers.

Categories	Functions
I	Conducting activity of muscle spindle
II	Conducting mechanical stimulation
III	Conducting algesia and thermesthesia
IV	Conducting algesia and thermesthesia
